# Placental abruption possibly due to parvovirus B19 infection

**DOI:** 10.1186/s40064-016-2946-2

**Published:** 2016-08-08

**Authors:** Ayaka Kawabe, Yasushi Takai, Jun-Ichi Tamaru, Kouki Samejima, Hiroyuki Seki

**Affiliations:** 1 Center for Maternal, Fetal and Neonatal Medicine, Saitama Medical Center, Saitama Medical University, 1981 Kamoda, Kawagoe, Saitama 350-8550 Japan; 2 Department of Obstetrics and Gynecology, Saitama Medical Center, Saitama Medical University, Kawagoe, Japan; 3 Department of Pathology, Saitama Medical Center, Saitama Medical University, 1981 Kamoda, Kawagoe, Saitama 350-8550 Japan

**Keywords:** Apoptosis, Human parvovirus B19, Neonatal asphyxia, Placental abruption, Pregnancy

## Abstract

**Background:**

There is concern about the development of anemia-associated fetal hydrops associated with maternal parvovirus B19 infection. Parvovirus B19 infection occurs via the globoside (P antigen) receptor, the main glycolipid of erythroid cells, which induces apoptosis. Similar findings have been reported for the P antigen of globoside-containing placental trophoblast cells.

**Case description:**

A 32-year-old woman was infected with human parvovirus B19 at week 32 of pregnancy, and had severe anemia at week 34. At week 37, an emergency cesarean section was performed because of sudden abdominal pain and fetal bradycardia; placental abruption was found. A live male infant was delivered with no sign of fetal hydrops or fetal infection. Placental tissue was positive for parvovirus B19 according to polymerase chain reaction. Immunohistochemical analysis using caspase-related M30 CytoDEATH monoclonal antibody revealed M30 staining of the placental villous trophoblasts.

**Discussion and evaluation:**

Placental trophoblasts and erythroid precursor cells have been reported to express globoside (P antigen), which is necessary for parvovirus B19 infectivity, and to show apoptotic activity as a result of infection. Placentas from three other pregnancies with documented abruption showed no M30 staining.

**Conclusion:**

The present case strongly suggests an association between placental abruption and apoptosis resulting from parvovirus B19 infection.

## Background

There is concern about the development of anemia-associated fetal hydrops associated with maternal parvovirus B19 (PB19) infection. PB19 infection occurs via the globoside (P antigen) receptor, the main glycolipid of erythroid cells, and induces apoptosis. Similar findings have been reported for the P antigen of globoside-containing placental trophoblast cells (Brown et al. 1993; Crane et al. 2014).

Here, we report a patient who developed placental abruption following severe anemia due to PB19 infection. Molecular pathologic examination of the placenta strongly suggested that PB19 infection induced apoptosis of the trophoblastic cells, leading to placental abruption.

This case study was approved by the Institutional Review Board of Saitama Medical Center, Saitama Medical University, and all subjects gave their consent for the use of their personal and medical information for the publication of this study.

## Case description

### A pregnancy with PB19 infection

This patient (case 1) was a 32-year-old woman who experienced two previous spontaneous vaginal deliveries without abnormality. She had a routine blood test at first and second trimester with unremarkable results. At approximately 32 weeks of pregnancy, both of her children had erythema infectiosum, and she developed a fever of about 38 °C at 34 weeks. Few days later, she started to complaine palpitations and dyspnea. She referred to our center at 36 weeks and 6 days of pregnancy due to continuous palpitations and severe anemia. When admission, no fetal disorder was found by ultrasonography; however maternal blood test results were as follows: white blood cell count, 5200/μL; hemoglobin, 5.3 g/dL; hematocrit, 15.1 %; platelets, 115,000/μL; segmented leukocytes, 84.0 %; monocytes, 4.0 %; lymphocytes, 12.0 %; reticulocytes, 1.8 %; and positive PB19 antibody IgM. Therefore, a diagnosis of pregnancy complicated with PB19 infection was made.

After emergency transfusion of eight units of red blood cells, her hemoglobin level recovered to 8.4 g/dL. At 37 weeks, a bone marrow biopsy revealed cytopenia, megaloblast, and a prominent decrease in erythroblast possibly due to PB19 infection. Therefore, she remained hospitalized for treatment of anemia.

**Fig. 1:**
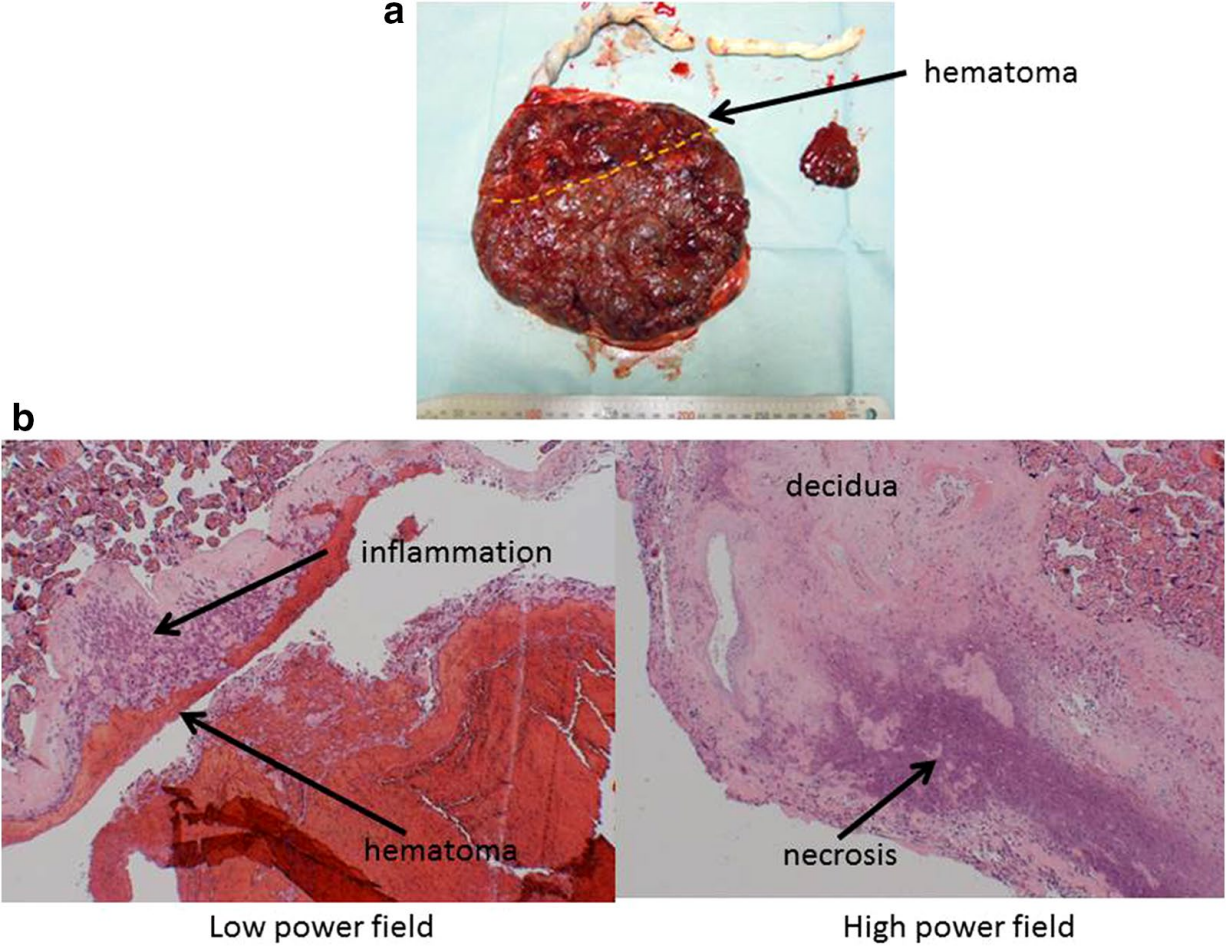
Macroscopic (**a**) and microscopic (**b**) findings of the placenta in the parvovirus B19 infection case. **a** Hematoma was found on approximately 30 % of the separated placenta. **b** Hematoxylin and eosin staining showed inflammation and necrosis localized to the decidua

At 37 weeks and 6 days, the patient experienced sudden abdominal pain, impaired consciousness, decreased blood pressure of 70/40 mmHg, and fetal bradycardia. Following rapid induction of general anesthesia, an emergency cesarean section was performed and a male infant of 2992 g was delivered. Apgar scores at 1 and 5 min were 1 and 4, respectively. Umbilical arterial pH was 6.781. During the surgery, hematoma was found on approximately 30 % of the separated placenta (Fig. 1a), leading to a diagnosis of placental abruption.

### Histopathologic finding of placenta

The diagnosis of placental abruption was consistent with the histopathologic examination, and hematoxylin and eosin staining showed inflammation and necrosis localized to the decidua (Fig. 1b). We hypothesized that PB19 induced apoptosis of the trophoblastic cells, leading to placental abruption. Accordingly, we performed a quantitative polymerase chain reaction (PCR) test (Umene and Nunoue 1995) for PB19-specific DNA to determine the presence of infection with microscopic sections of placenta and confirmed apoptosis of the decidual and chorionic sites using immunostaining with M30 CytoDEATH antibody (M30 Mab; VLVbio AB, Stockholm, Sweden), which identifies cytokeratin 18 fragmented due to apoptosis (Jordan and Butchko 2002; Abumaree et al. 2012).

To rule out a possible false-positive reaction, the above procedures also were performed on placental sections obtained from three other control cases (case 2,3,4), who were clinically and histopathologically diagnosed with placental abruption without PB19 infection at our center between January 2009 and December 2011 (Table 1). The results were as follows.

**Table 1:** Demographics of control cases with placental abruption

Case No.	Age, y	Gravida, para	Complication(s)	Length of hospitalization for this pregnancy	Delivery	Infant characteristics and Page’s classification for placental abruption
2	33	3, 0 3 SA	Myoma after uterine septectomy	12–13 weeks, threatened abortion After 26 weeks, premature delivery	30 weeks 4 days, CS	Birth weight 1546 g Apgar score 6/7^a^ UApH level 7.336 Grade 1 placental abruption
3	34	1, 1 PROM at 25 weeks ⇒ neonatal death	Myoma endometrial cyst after FET	10–13 weeks, subchorionic hematoma 16–20 weeks, threatened abortion	24 weeks 0 days, CS	Birth weight 526 g Apgar score 3/8^a^ Grade 1 placental abruption
4	32	1, 1	PIH	–	38 weeks 1 day, CS	Birth weight 2642 g Intrauterine fetal death Grade 2–3 placental abruption

*CS* cesarean section, *FET* frozen embryo transfer, *PIH* pregnancy-induced hypertension, *UApH* umbilical artery pH, *PROM* premature rupture of the membranes, *SA* spontaneous abortion

^a^Apgar scores at 1 and 5 min (1/5)

**Fig. 2:**
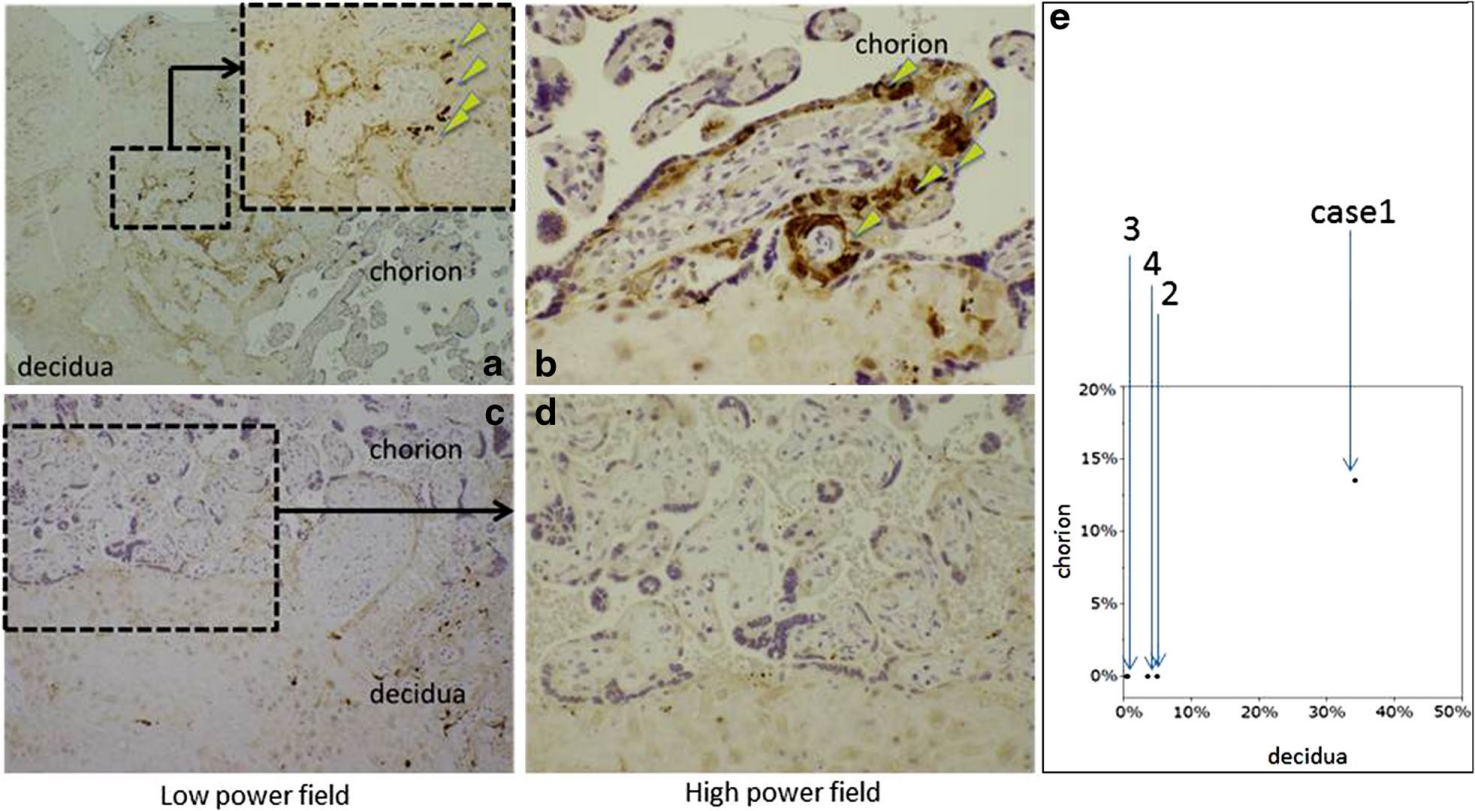
Immunohistochemical findings of all placentas using M30 CytoDEATH antibody. Immunostaining for apoptosis in case 1 **a, b** showed positive findings in the decidual and contiguous chorionic cells (*arrows*), while the three control cases, including case 2 **c, d**, showed no staining of the chorionic cells. **e** Graph, showing the percentage of M30-positive decidual and chorionic cells in each case

In case 1, more than 10^5^ copies of PB19-specific DNA were detected, while all three control cases were negative, suggesting that case 1 was not a false positive (data not shown). Immunostaining for apoptosis was positive in 34.2 % of the decidual cells and in 13.6 % of the chorionic cells. In contrast, cases 2, 3, and 4 showed positive immunostaining of small numbers of decidual cells (4.9, 0.5, and 3.6 % respectively), and no staining of chorionic cells (Fig. 2). For quantitative evaluation, five fields with apparent positive findings from each section of the placenta in all cases were extracted at 200-fold magnification, and the percentages of stained cell nuclear were calculated. Indistinct or faintly-stained areas were excluded.

### Patient’s follow up

Neonatal blood test showed a level of 16.9 g/dL of hemoglobin, 3.2 % of reticulocytes and negative PB19 IgM. The baby was diagnosed with severe neonatal asphyxia and underwent brain hypothermia therapy, but developed neither fetal hydrops nor fetal anemia. He didn’t have PB19 infection. Both mother and baby had good course, and now 3 years old, the baby has experienced no abnormality of growth or development.

## Discussion and evaluation

Histlogical findings of placenta suggested that PB19-specific DNA were present and apoptosis was almost exclusively observed to a greater extent in the chorionic and decidual cells. These findings were consistent with our hypothesis that placental abruption was caused by apoptosis of the chorion and decidua due to PB19 infection.

In pregnant women, placental infection with PB19 is considered problematic. Therefore, it has been recommended that PB19-infected pregnant women undergo serial ultrasound including fetal middle cerebral Doppler every 1–2 weeks to check for any abnormalities, such as fetal anemia and fetal hydrops (Crane et al. 2014; Minakami et al. 2014). Prevalence of PB19 immunoglobulin G is 50–75 % in women of reproductive age in Europe and the USA (Crane et al. 2014), and slightly under 50 % in Japanese adults (unpublished data). PB19 infection is rarely severe in adults, and many cases present with nonspecific symptoms, such as fever, arthralgia, or minor exanthems, even in an initial infection. Consequently, it is difficult to diagnose and manage PB19 infection at an early stage unless the mother or child shows an abnormality.

Fetal hydrops and cardiac enlargement are commonly detected on ultrasonography. P antigen, which is considered to be the receptor for PB19, is found to be a globoside, a neutral glycolipid that accumulates in red blood cell membrane lipid rafts. When PB19 binds to P antigen, apoptosis is induced. P antigen is expressed in a variety of cells, including placental thromboblastin (Brown et al. 1993).

Although case 1 was an adult with an initial PB19 infection, no fetal disorder was found; the mother exhibited severe anemia and slightly decreased blood platelets, but her anemia did not worsen after transfusion. She developed placental abruption during hospitalization, however, she had no risk factors for abruption (Cunningham et al. 2014; Oyelese and Ananth 2006).

In Japan, up to 60 % of placental abruption cases do not exhibit known risk factors. However, it has been reported that chorioamnionitis and apoptosis are associated with preterm placental abruption. Apoptosis of the trophoblasts leads to necrosis and/or angionecrosis of the chorion and amnion, and promotes production of prostaglandins; this enhances uterine contractions, resulting in placental abruption caused by the gap between the uterus and placenta. In addition, macrophages and dendritic cells phagocytize necrotic trophoblasts and release cytokines, causing chorioamnionitis, which leads to enhanced uterine contractions, ultimately resulting in placental abruption (Oyelese and Ananth 2006; Tikkanen et al. 2010; Nakatsuka et al. 1999; Nath et al. 2007). Therefore, we thought that it was very likely that PB19 infection resulted in abruption.

There has been no report similar to the present case. Consequently, to clarify the causal relationship between maternal PB19 infection and placental abruption, further study is needed. For example, detection of apoptosis using immunostaining with M30 Mab should be performed as much as possible in women potentially infected with PB19, including those who have experienced miscarriage or stillbirth. We anticipate comparing these PB19-related cases with or without placental abruption, and will perform further studies to determine the differences in histologic findings of the placenta in order to clarify the cause and course of placental abruption. Additionally, more substantial information might have been needed for further discussion (e.g. viral load in maternal plasma and immunohistochemical detection of PB19 antigen).

We report this case because maternal complications such as placental abruption might develop even if fetal health is good. With the assumption that placental abruption may be caused by apoptosis in the chorion in mothers with severe PB19 infection, clinical management may require high-level intervention equivalent to that for preterm premature rupture of the membranes and chorioamnionitis.

## Conclusion

In the present case, apoptosis of the trophoblastic cells due to PB19 infection might have resulted in placental abruption. Mothers with severe PB19 infection may require high-level intervention equivalent to that for preterm premature rupture of the membranes and chorioamnionitis even if their fetus show little sign of hydrops fetalis. To clarify the association between maternal PB19 infection and placental abruption, further study is needed.

## Abbreviations

PB19: human parvovirus B19
PCR: polymerase chain reaction
CS: cesarean section
FET: frozen embryo transfer
PIH: pregnancy-induced hypertension
UApH: umbilical artery pH
PROM: premature rupture of the membranes
SA: spontaneous abortion
